# Falls prevention advice and visual feedback to those at risk of falling: study protocol for a pilot randomized controlled trial

**DOI:** 10.1186/1745-6215-14-79

**Published:** 2013-03-19

**Authors:** Stephen Uzor, Lynne Baillie, Dawn A Skelton, Phillip J Rowe

**Affiliations:** 1 Glasgow Caledonian University, Glasgow, United Kingdom; 2 University of Strathclyde, Glasgow, United Kingdom

**Keywords:** Falls, Rehabilitation, Games, Visualization, Inertial sensors, RCT

## Abstract

**Background:**

Studies have shown that functional strength and balance exercises can reduce the risk of falling in older people if they are done on a regular basis. However, the repetitive nature of these exercises; combined with the inherent lack of feedback of progress may discourage seniors from exercising in the home, thereby rendering such an intervention ineffective. This study hypothesizes that the use of visual feedback and multimodal games will be more effective in encouraging adherence to home rehabilitation than standard care; thereby promoting independence and improving the quality of life in older adults at risk of falling.

**Methods:**

A pllel-group pilot randomized controlled trial with 3 groups of participants will be conducted in the home for 12 weeks. Participants will include older adults who have been identified as at risk of falling (n = 48), over the age of 65, living in the community, and suitable for a home exercise intervention. The primary outcome is adherence to exercise. Secondary outcomes include: variability in stride length, stride time and double support time (DST); walking speed; Timed up and go test (TUG); Falls Efficacy Scale International (FES-I); CONFbal scale; Romberg’s test; and quality of life measures (SF-12 and EuroQol EQ-5D). Qualitative assessments on personal experiences with rehabilitation tools will be done before and after the trial.

**Discussion:**

This study will investigate the use of visual feedback and engaging multimodal activities to address the problem of non-compliance to home exercises for falls rehabilitation. One of the unique qualities of this study is the adaptation of special participatory design methods through which the end users (fallers) will be involved in the design of the proposed rehabilitation tools at various stages of the design process.

**Trial registration:**

ISRCTN79967470

## Background

### Falls and adherence to exercise

Thirty percent of people over the age of 65 years fall at least once a year [1], with over 75% of this population suffering an injury as a result. Fractures (a major consequence of falls) alone cost the National Health Service (NHS) in the UK about £1.8 billion [2]. Although falls can be caused by a wide variety of factors (including visual and physical impairment), many cases are attributed to a lack of exercise as well as weaknesses in muscles, bones and joints in the lower body [3]. These conditions can be improved by rehabilitation designed to improve the patient’s ability to cope with movement in everyday life, increase the patient’s functional capacity, elevate confidence and reduce the fear of falling [4].

Evidence has shown that certain rehabilitation exercises such as the Otago exercise program and Falls Management Exercise (FaME) are effective in returning falls patients to normal functional movement if the patient adheres to the routine [5]. However, there is an existing problem with adherence to these exercises, which could be attributed to a wide variety of factors, such as low confidence in the rehabilitation programme and lack of motivation on the user’s part [6]. In the home, motivation to exercise can also be affected by the somewhat repetitive nature of the current exercises and the way that they are presented to the user (through paper-based illustrations), which could make the rehabilitation process seem like a chore for the patient [7,8].

To address this problem of low adherence to home exercises, it is necessary to first identify the common problems that fallers face in the home, and what effects these problems may have on seniors’ confidence, fear of falling, and quality of life. Furthermore, it is essential to explore the limitations of standard rehabilitation care in order to determine how these limitations may affect their adherence to home rehabilitation programmes. In this study, there is an investigation into how visualizations and games could be used to encourage greater participation in home rehabilitation.

### Visual feedback of activity

In the past, visualizations of movement have been explored by Macdonald *et al*. [9] to promote engagement in rehabilitation. The theory behind this study suggested that if the seniors were more involved in their rehabilitation, they could take greater steps to ensure that they became more independent. This study involved testing gait in the lower limbs, while providing visualizations of the exercise using a motion capture system. The results showed that the simple visualizations (which consisted of a stick figure on a screen using a traffic light system - green, amber and red - to show varying levels of stress in joints during activity at different times) were able to bridge the gap in the understanding of important data between the patient, clinicians, biomechanists and physiotherapists. By doing this, the seniors felt more involved because a discussion could be had between them and the therapists on how best to facilitate their recovery. In a similar vein, the study by Yavuzer *et al*. [10] revealed how the use of visual feedback through balance training, in addition to a conventional stroke rehabilitation programme, improved postural control and weight-bearing in late-stage stroke patients. One of the limitations of these studies is that factors such as equipment size, cost, and the complex setup involved means that the experiments need to be restricted to the clinical, or laboratory setting.

Virtual environments and video games have also proved successful in providing an engaging rehabilitation experience for patients with a wide variety of ailments, including stroke and chronic obstructive pulmonary disease (COPD) [11,12]. The use of commercially available games such as Wii-Fit has become a staple of encouraging physical activity in older adults, mostly in the clinical setting [13,14]. However, rehabilitation often involves specifically tailored exercises that promote the recovery of muscle groups necessary for improved strength, balance and mobility. Therefore, there is a need to go one step further in the design of games, in order to make them useful for rehabilitation purposes. It is believed that an efficient way to tackle this problem is to design games that encourage limb movements that are characteristic of rehabilitation, in order to promote effective recovery of muscle strength and balance.

In one arm of the study described in this paper, we will explore how visualizations of movement may be used to encourage older adults at risk of falling, to exercise in the home. The main aim of the visualizations in this study will be to advise users on the most efficient way to perform each exercise. Using simple body-worn sensors, the users’ body movements can be tracked, and the information from these sensors can be used to inform the users on simple biomechanical concepts that are useful to their rehabilitation, such as joint angles and range of motion.

To tackle the ever-present problem of the lack of interest and adherence to the rehabilitation routine, this study also aims to employ the use of multimodal games, based on rehabilitation exercises, which will enable users to perform exercises in an enjoyable manner and hence provide a suitable opportunity for effective functional recovery. To maximize the potential of the use of games for this purpose, older adults have been involved at various stages of the design process, including the initial concept design stage, in order to address the main issues that they may face, through the use of these tools in a realistic unsupervised home setting [8]. It is anticipated that by using games to exercise, the repetitive movements necessary for rehabilitation can be made more enjoyable for the user, which should have a positive effect on confidence and adherence to the exercise programmes in the home.

## Methods/design

### Research questions

1. Can visual feedback of movement during home exercise improve adherence to falls rehabilitation programmes?

2. Can the use of multimodal games for falls rehabilitation in the home improve adherence to exercise?

### Study design

**Figure 1:**
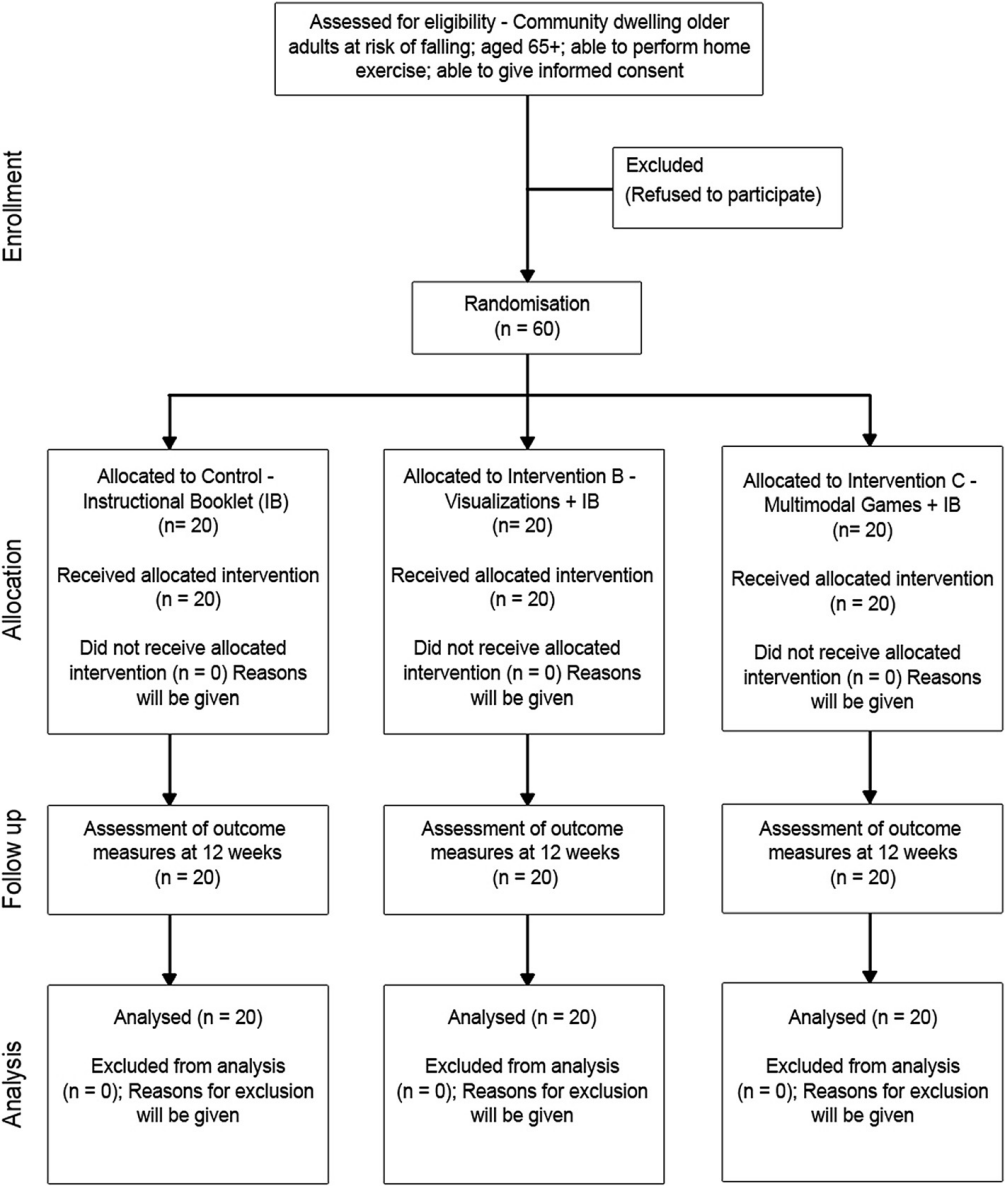
Flow diagram of trial design showing the three arms of the study (control, visualizations and multimodal games).

The study will involve a three-arm, pllel-group randomized controlled trial (RCT) based in the community. Participants will provide written informed consent prior to taking part in the study. Randomization will be done using a computer-based number generator. Participants in all three groups will be allocated an intervention based on a 1:1:1 ratio (Figure 1). The trial will be carried out over a 12-week period.

### Home-based falls rehabilitation

Home rehabilitation for falls includes specially tailored exercises designed to improve muscle strength and balance in the lower limbs, as well as core stability. This is achieved by challenging important muscles, as well as training joints through their normal range of movement for a set number of repetitions (or for a certain amount of time). For falls rehabilitation, these exercises are usually provided to older adults in the form of instructional booklets or videos. Each exercise session involving the use of these tools is expected to last for 30 to 45 minutes.

### Intervention

The Age UK falls rehabilitation booklet [15] is commonly used in falls services in the UK for home-based exercise and will be used as standard care in this study. This booklet contains a warm-up, a cool-down and ten exercises in total, comprising five balance and five strength exercises. Following recruitment, the participants will be allocated to one of three interventions in this study, namely control, visualization, or multimodal games.

Control: the control group will receive the standard home-based rehabilitation care for falls prevention provided by the NHS Greater Glasgow and Clyde Falls Service. This includes the Age UK booklet previously mentioned.

Visualization: the participants in this group will receive visualizations of their movement during home rehabilitation in addition to standard care. The visualizations will serve three main purposes as follows: to 1) present the rehabilitation exercises to the participants using an animated mannequin that will demonstrate the correct range and speed of motion required for each exercise. Instructions on how to perform these exercises will also be shown; 2) show the users real-time feedback of their movements as they perform their exercises and provide a summary of their performance during the process, and 3) provide lay information on the principles behind strength and balance exercises, and to explain the role of these exercises in the prevention of falls.

**Figure 2:**
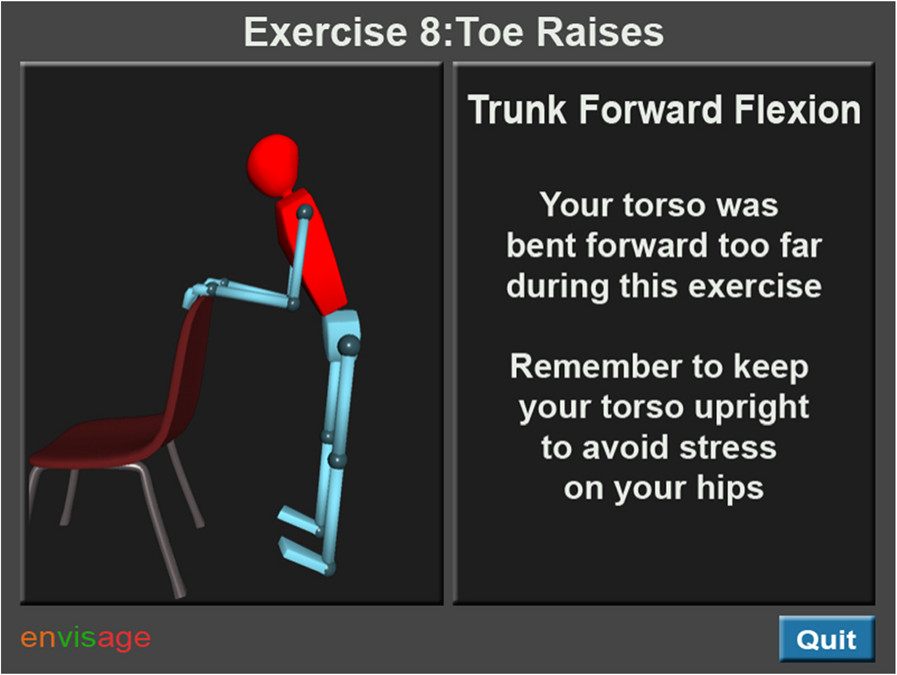
Concept design of visualizations showing users when they performed exercises incorrectly.

The proposed visualization tool will be able to execute these functions in a variety of ways. First, through the use of a digital mannequin, the users will receive information on the ideal way to perform strength and balance exercises. The goal of falls rehabilitation is to perform exercises using a recommended (by a physiotherapist) range and speed of motion. By performing these movements using affected limbs, strength can be restored, or acquired, in the relevant limb/body segment(s). We have collaborated with leading falls experts and physiotherapists in the local falls service on the definition and incorporation of these optimal movements into our visualization software. Second, the users will have the ability to visualize their own movements on a screen, using input from body-worn inertial sensors. This is to enable them to compare their movements with the recommended movement. Third, the visualization tool will provide users with a summary of their movement performance, and advise them on how to avoid and correct any incorrect/compensatory movements that occur during home exercise sessions (Figure 2).

Multimodal games: participants in this group will receive multimodal games in addition to the standard care during the trial. Multimodal games can be used to encourage users to exercise, as the use of games provides a sense of engagement and distracts the user from the essential but repetitive nature of rehabilitation exercises. The games will serve two main functions: to 1) provide the users with a more interactive and enjoyable way to perform home exercise, and 2) inform the users on their performance and progress during rehabilitation by tracking game scores, achievements and correct repetitions of movements during exercise.

To ensure that the games satisfy the requirements necessary for effective falls rehabilitation, their main mechanics will be based on movements required to successfully perform the exercises in the Age UK booklet. The users will be able to perform their exercises by interacting with the games through full body interaction, using inertial sensors strapped to their arms and legs.

**Figure 3:**
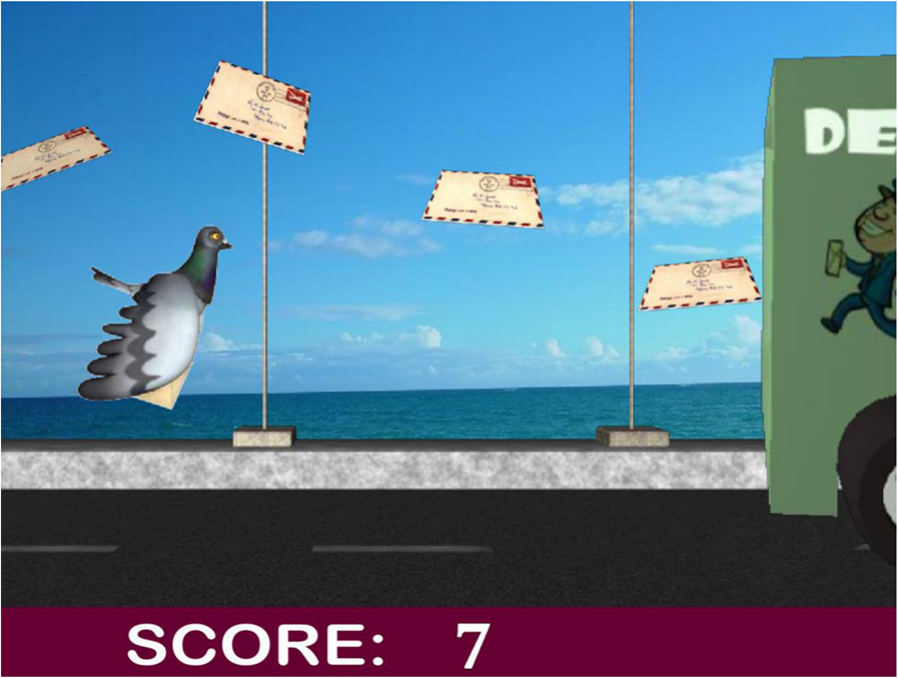
Early design for a game based on the sit-to-stand exercise.

When it comes to designing games and other multimodal activities for rehabilitation, we believe that it is not enough for young designers to decide what older adults will consider an enjoyable experience. Previous studies in this field have recognized the need to evaluate such tools with older adults, especially in stroke rehabilitation [16], but have stopped short of actually encouraging the seniors to design their own tools. This study aims to include users in the design of games in order to address many of the concerns that they may have regarding the use and interaction with the tools. Figure 3 shows one of the games co-designed with older adults at a user workshop conducted as part of this research. The base mechanics of this game (which involved users having to sit and stand to control the flight of a bird in order to collect letters in the game) were suggested by seniors at a workshop, where they worked in groups to design various games for the different falls exercises [8]. Pre-trial pilot studies with fallers will also ensure that the games are designed and modified to a suitable specification for use in the trial.

### Hypothesis

The home-based use of visualizations and multimodal games will be more effective for encouraging adherence to home rehabilitation programmes than standard care, thereby improving adherence to exercise, confidence and quality of life.

### Participants

Participants who have had injurious falls will be recruited from the NHS Greater Glasgow and Clyde community falls prevention programme (CFPP). Participants who have had non-injurious falls will be recruited from the Glasgow Housing Association Sheltered Housing Scheme. Recruitment will occur at the point where older adults at risk of falling are offered home exercise by the physiotherapists at the CFPP, who will hand out information sheets and explain the study to the potential participants. Interested participants who meet the inclusion criteria and have given informed consent will then be randomized into one of the groups involved in the trial.

### Sample size

This study is a pilot RCT and as such, no power calculation has been carried out. It is intended that the results obtained from this study will inform the design of a larger trial upon which a robust power calculation can be done based on adherence to exercise.

### Inclusion criteria

Participants will be included in the study if they are over the age of 65 years; have had a fall in the past 12 months; live in the community; are able to perform home exercise; are able to give informed consent, and can understand simple instructions using the English language (to understand instructions from visualizations/games).

### Exclusion criteria

Participants will be excluded from the study if they are unable to give informed consent; have major cognitive visual or aural impairments, or are unable to understand instructions in English (necessary to understand the instructions from the visualizations/multimodal games). Exclusion criteria 1 and 2 will be determined with assistance from the physiotherapists and falls experts within the falls service and from Scheme managers in the Sheltered Housing Schemes.

### Outcomes

The outcomes for this study are consistent with useful relevant studies in falls prevention. They include measures of compliance, and gait/balance performance, as well as the use of questionnaire-based health and quality of life evaluation tools, which provide a multi-factorial assessment of a person’s falls risk. All outcome measures will be assessed at the start and at the end of the 12-week trial.

#### Primary outcome measure

##### Adherence to exercise

All of the participants will be required to record their exercise sessions on paper using a series of tick boxes, detailing what exercises they did, how many times they did them, and on what day they did the exercises. The sensors used in the intervention groups will automatically log usage data, which will reflect the amount of exercise performed.

#### Secondary outcome measures

##### Variability in stride length, stride time and double support time (DST)

Stride length and stride time are measures associated with the distance between one foot at the start and at the end of one gait cycle, and the time taken to complete this cycle. DST is the amount of time that both feet are simultaneously planted on the ground, and is expected to be 10% of one gait cycle [17]. The stride-to-stride variability in these measures has been shown to be a major predictor of falls risk in the elderly [18]. These measures will be captured using the GAITRite system [19].

##### Gait speed

This measure indicates how fast a person walks, and it is known to be associated with falls risk. For instance, there is a suspected higher risk of outdoor falls in fast walkers, and a higher risk of indoor falls in slow walkers. Gait speed in slow walkers is expected to improve with regular exercise [20-22] and will be captured using the GAITRite system.

##### Timed up and go test (TUG)

The TUG test is one of the most commonly used clinical measures for assessing gait and balance in older patients. Patients rise from a chair, walk a distance of 3 metres, and then return to the chair while being timed with a stopwatch. Healthy older adults are expected to complete the task in less than 10 seconds. A time >14 seconds is known to be associated with a high risk of falling [20,23].

##### Romberg’s test

Romberg’s test is a standing balance test used to diagnose proprioceptive, vestibular and visual disorders related to maintaining balance. Patients are required to stand with their feet together, eyes open and their hands by their sides for a period of time, usually a minute. The process can then be repeated with their eyes closed [24]. A positive Romberg sign is issued when the subject fails to maintain the standing posture during these tests.

##### *The measure of balance confidence*(*CONFBal*) *scale*

The CONFbal scale is a 10-item scale used to assess self-efficacy and confidence in maintaining balance [25,26]. Scores to the individual questions are assessed on a scale of 1 to 3 (1 = confident, 3 = not confident).

##### Falls efficacy scale international (FES-I)

The FES-I is a useful tool that measures physical and social activities, as well as the level of concern about falling during these activities, both indoors and outdoors. The level of concern is measured on a 4-point Likert scale (1 = not at all concerned,4 = very concerned) [27-29].

##### Quality of life - EuroQOL (EQ)-5D and Short Form (SF)-12

These are useful commonly used questionnaire-based tools for the assessment of quality of life [30-35].

### Equipment

#### Home use

This study will utilize small inertial sensors (developed by Glasgow Caledonian University) as the primary control mechanism for the software tools. These inertial sensors, which provide a relatively cheap, and accurate motion capture system, will be strapped to various limbs depending on the exercise required, and will translate the users’ movements into the relevant motion in the software. During the trial, user feedback through visualizations of movement, or games will be shown to the user on a television connected to a laptop computer.

#### Home assessment and training

The participants will be visited at the start of the trial by the chief investigator and a research nurse, who are both trained in environmental risk assessment. During this visit, the chief investigator will train the participants how to perform exercises using the visual feedback and games systems. Through usability studies that will be conducted prior to the start of the trial, it is intended that the best possible way to keep the training time to a minimum will be identified. This means that the visualization and games systems will be designed to be accessible and easy to use. The participants will be asked to perform an exercise session during the home visit to ensure that they understand how to use the systems properly. A handbook on the operation of the system will also be provided to the participants, with clear annotated diagrams highlighting the various components, as well as details on achieving the maximum benefits offered by the system. Furthermore, the participants’ homes will be assessed to ensure that there is enough space to perform the exercises in a safe manner. Both the chief investigator and the research nurse will also assess the environment in order to identify an appropriate location for the equipment (laptop computer and inertial sensors) through which the participants will interact with the visualization/games.

### Statistical analysis

For qualitative data, Likert scales will be used for some questions, and for the open-ended questions, thematic coding will be used. Quantitative data from the laboratory tests (baseline and outcome measures of gait and balance) will be analysed on an intention-to-treat basis. Statistical analyses will focus on the possible existence of differences in outcomes between the control and intervention groups using a two-way, repeated-measures analysis of variance (ANOVA).

### Ethical approval

Ethical approval for this study was granted by the NHS Research Ethics Committee 3, Scotland, UK (Reference: 11/AL/0383).

## Discussion

A problem with adherence to exercise has been identified in community falls prevention as well as in research trials, and this is the basis for this study. Participants included in this study will be provided with tools that could potentially increase their motivation to perform home exercise, which in turn could help improve their gait, balance and mobility. In addition, the use of these tools could reduce their fear of falling (if present) and improve their quality of life. The multimodal games and visualizations also have the potential to help the participants understand why each of the exercises is important to their recovery and to assure them of the progress that they make during their rehabilitation.

The use of computers and inertial sensor technologies is relatively new to older adults at risk of falling (age 65 years and older). It was necessary to identify the major problems that they may face when using such technologies in the absence of a professional. Some of the most important barriers may include using the computer, strapping on and managing the inertial sensors (for example, charging the sensors), and successfully navigating their way around the software. In this project, seniors have been involved in the initial design and development of the visualizations, games and sensors. This user involvement process included: 1) involving older adults in design workshops to identify the limitations of standard home rehabilitation care (booklets); 2) determination of the requirements necessary to ensure that the proposed intervention tools were useful to them (visualizations and games), and 3) generation of concept ideas for the appearance and operation of these tools [7,8]. It is the intention of this project to involve elderly users in the iterative design and modification of these tools in usability studies prior to the start of the randomized controlled trial. This will be done in order to ensure that these relatively new technologies are easy to use and manage by older adults in the home.

## Trial status

Recruitment for the trial started in October 2012, and will end in July 2013.

## Abbreviations

ANOVA: Analysis of variance; CFPP: Community Falls Prevention Programme; CONFBal: Measure of balance confidence; COPD: Chronic obstructive pulmonary disease; DST: Double support time; EQ: EuroQOL; FaME: Falls management exercise; FES-I: Falls efficacy scale international; NHS: National Health Service; RCT: Randomized controlled trial; SF-12: Short Form-12; TUG: Timed up and go.

## Competing interests

The authors declare they have no competing interests.

## Authors’ contributions

To date, SU has had the responsibility of preparing this manuscript and the study protocol. LB, DS and PR have played significant roles in the development, critical assessment, and modification of the procedures detailed in this document, and they have read and approved this manuscript in its current form.

## References

[B1] Todd C Skelton D WHO regional office for Europe 2004 Copenhagen: Health Evidence Network report http://www.euro.who.int/document/E82552.pdf] [Accessed 5th October 2012

[B2] Oliver D Development of services for older patients with falls and fractures in England: successes, failures, lessons and controversies Arch Gerontol Geriatr 2009 49 Suppl 2 S7 S12 20005429 10.1016/S0167-4943(09)70005-6

[B3] Rubenstein LZ Falls in older people: epidemiology, risk factors and strategies for prevention Age Ageing 2006 35 suppl 2 ii37 ii41 16926202 10.1093/ageing/afl084

[B4] Skelton DA Dinan SM Exercise for falls management: rationale for an exercise programme aimed at reducing postural instability Physiother Theory Pract 1999 15 105 120 10.1080/095939899307801

[B5] Sherrington C Tiedemann A Fairhall N Close JCT Lord SR Exercise to prevent falls in older adults: an updated meta-analysis and best practice recommendations NSW Public Health Bull 2011 23 78 83 10.1071/NB10056 21632004

[B6] Nyman SR Victor CR Older people’s recruitment, sustained participation, and adherence to falls prevention interventions in institutional settings: a supplement to the Cochrane systematic review Age Ageing 2011 40 430 436 10.1093/ageing/afr016 21502163

[B7] Uzor S Baillie L Skelton DA Fairlie F Campos P, Graham N, Jorge J, Nunes N, Palanque P, Winckler M Identifying Barriers to Effective User Interaction with Rehabilitation Tools in the Home Human-Computer Interaction (INTERACT) 2011 Springer Berlin Heidelberg 36 43

[B8] Uzor S Baillie L Skelton DA Senior designers: empowering seniors to design enjoyable falls rehabilitation tools Proceedings of the thirty-first ACM SIGCHI conference on computer human interaction (CHI) 2012 ACM Press 1179 1188

[B9] Macdonald AS Loudon D Innovation in envisioning dynamic biomechanical data to inform healthcare and design guidelines and strategy 2012 Sheffield: NDA Research Programme http://www.newdynamics.group.shef.ac.uk/assets/files/172.pdf

[B10] Yavuzer G Eser F Karakus D Karaoglan B Stam HJ The effects of balance training on gait late after stroke: a randomized controlled trial Clin Rehabil 2006 20 960 969 10.1177/0269215506070315 17065539

[B11] Alankus G Lazar A May M Kelleher C Towards customizable games for stroke rehabilitation Proceedings of the twenty-eighth ACM SIGCHI conference on computer human interaction (CHI) 2010 ACM Press 2113 2122

[B12] Sveistrup H Motor rehabilitation using virtual reality NeuroEng Rehab 2004 1 10 10.1186/1743-0003-1-10 PMC546406 15679945

[B13] Sugarman H Weisel-Eichler A Burstin A Brown R Use of the Wii Fit system for the treatment of balance problems in the elderly: a feasibility study 2009 IEEE: In Proceedings of the virtual rehabilitation conference 111 116

[B14] Deutsch JE Robbins D Morrison J Bowlby PG Wii-based compared to standard of care balance and mobility rehabilitation for two individuals post-stroke Proceedings of the international conference on virtual rehabilitation (ICVR) 2009: IEEE 117 120

[B15] Age UK, Preventing Falls: Strength and balance exercises for healthy ageing 2012 http://www.ageuk.org.uk/Documents/EN-GB/ID8950%20Strength%20And%20Balance%20Book.pdf?dtrk = true

[B16] Burke JW McNeill MDJ Charles DK Morrow PJ Crosbie JH McDonough SM Optimising engagement for stroke rehabilitation using serious games Proceedings of the First International IEEE Conference on Serious Games and Virtual Worlds (VS Games) 2009 12: The Visual Computer, Springer 1085 1099

[B17] Khodadadeh S McClelland MR Nene AV Patrick JH The use of double support time for monitoring the gait of muscular dystrophy patients Clin Biomech 1987 2 68 70 10.1016/0268-0033(87)90129-X 23915646

[B18] Hausdorff J Gait dynamics, fractals and falls: finding meaning in the stride-to-stride fluctuations of human walking Hum Mov Sci 2007 26 555 589 10.1016/j.humov.2007.05.003 17618701 PMC2267927

[B19] Van Uden CJT Besser MP Test-retest reliability of temporal and spatial gait characteristics measured with an instrumented walkway system (GAITRite®) BMC Musculoskelet Disord 2004 5 13 10.1186/1471-2474-5-13 15147583 PMC420245

[B20] Viccaro LJ Perera S Studenski SA Is timed up and go better than gait speed in predicting health, function, and falls in older adults? Am Geriatrics Soc 2011 59 887 892 10.1111/j.1532-5415.2011.03336.x PMC3522463 21410448

[B21] Quach L Galica AM Jones RN Procter-Gray E Manor B Hannan MT Lipsitz LA The nonlinear relationship between gait speed and falls: the maintenance of balance, independent living, intellect, and zest in the elderly of Boston study J Am Geriatr Soc 2011 59 1069 1073 10.1111/j.1532-5415.2011.03408.x 21649615 PMC3141220

[B22] Judge JO Underwood M Gennosa T Exercise to improve gait velocity in older persons Arch Phys Med Rehabil 1993 74 400 406 8466422

[B23] Shumway-Cook A Brauer S Woollacott M Predicting the probability for falls in community-dwelling older adults using the timed up & go test Phys Ther 2000 80 896 903 10960937

[B24] Khasnis A Gokula RM Romberg’s test J Postgrad Med 2003 49 169 172 12867698

[B25] Simpson JM Worsfold C Hawke J Balance confidence in elderly people: the CONFbal scale. [abstract] Age Ageing 1998 27 Suppl 2 57

[B26] Simpson JM Worsfold C Fisher KD Valentine JD The CONFBAL scale: a measure of balance confidence – a key outcome of rehabilitation Physiotherapy 2009 95 103 109 10.1016/j.physio.2008.12.004 19627691

[B27] Yardley L Beyer N Hauer K Kempen G Piot-Ziegler C Todd C Development and initial validation of the falls efficacy scale-international (FES-I) Age Ageing 2005 34 614 619 10.1093/ageing/afi196 16267188

[B28] Hauer K Yardley L Beyer N Kempen G Dias N Campbell M Becker C Todd C Validation of the falls efficacy scale and falls efficacy scale international in geriatric patients with and without cognitive impairment: results of self-report and interview-based questionnaires Gerontology 2010 56 190 199 10.1159/000236027 19729878

[B29] Kempen GIJ Yardley L van Haastregt JCM Zijlstra GA Beyer N Hauer K Todd C The short FES-I: a shortened version of the falls efficacy scale-international to assess fear of falling Age Ageing 2008 37 45 50 18032400 10.1093/ageing/afm157

[B30] König HH Ulshöfer A Gregor M von Tirpitz C Reinshagen M Adler G Leidl R Validation of the EuroQol questionnaire in patients with inflammatory bowel disease Eur J Gastroenterol Hepatol 2002 14 1205 1215 10.1097/00042737-200211000-00008 12439115

[B31] Ellis JJ Eagle KA Kline-Rogers EM Erickson SR Validation of the EQ-5D in patients with a history of acute coronary syndrome Curr Med Res Opin 2005 21 1209 1216 10.1185/030079905X56349 16083530

[B32] Gandek B Ware JE Aaronson NK Apolone G Bjorner JB Brazier JE Bullinger M Kaasa S Leplege A Prieto L Sullivan M Cross-validation of item selection and scoring for the SF-12 health survey in nine countries: results from the IQOLA project. International quality of life assessment J Clin Epidemiol 1998 51 1171 1178 10.1016/S0895-4356(98)00109-7 9817135

[B33] Resnick B Nahm ES Reliability and validity testing of the revised 12-item short-form health survey in older adults J Nurs Meas 2001 9 151 161 11696939

[B34] Ozcan A Donat H Gelecek N Ozdirenc M Karadibak D The relationship between risk factors for falling and the quality of life in older adults BMC Publ Health 2005 5 90 10.1186/1471-2458-5-90 PMC1208910 16124871

[B35] The SF12: An even shorter health survey http://www.sf36.org/tools/sf12.shtml] [Accessed 5th October 2012

